# Peripheral microvascular function and in-stent late lumen loss after percutaneous coronary intervention: a retrospective cohort study with an exploratory analysis by implanted stent diameter

**DOI:** 10.3389/fcvm.2026.1940897

**Published:** 2026-10-27

**Authors:** Hangyeol Kim, Hyun Sung Joh, Woo-Hyun Lim, Hack-Lyoung Kim, Sang-Hyun Kim, Jae-Bin Seo

**Affiliations:** 1 Department of Internal Medicine, Seoul National University Hospital, Seoul National University College of Medicine, Seoul, Republic of Korea; 2 Cardiovascular Center, Seoul National University Boramae Medical Center, Seoul, Republic of Korea

**Keywords:** endothelial function, in-stent restenosis, late lumen loss, peripheral arterial tonometry, quantitative coronary angiography, reactive hyperemia index, stent diameter

## Abstract

**Introduction:**

Peripheral microvascular endothelial function measured by the reactive hyperemia index (RHI) with EndoPAT predicts cardiovascular outcomes, and post-procedural RHI has been associated with binary in-stent restenosis. Whether pre-procedural RHI relates to in-stent late lumen loss (LLL) measured by quantitative coronary angiography (QCA) after percutaneous coronary intervention (PCI), and whether any association depends on device caliber, has not been established.

**Methods:**

We retrospectively analyzed 132 patients who underwent EndoPAT before PCI (index PCI 2009-2016). The primary endpoint was in-stent LLL at follow-up angiography (*n* = 91; median follow-up 9.4 months). Because in-stent LLL was highly skewed, the primary analysis used Spearman correlation and a robust (HC3) multivariable regression adjusting for age, diabetes, hypertension, number of diseased vessels, and pre-PCI minimal lumen diameter. Pre-specified sensitivity analyses included multiple imputation (MICE, m = 20) for missing follow-up and lesion-level generalized estimating equations (GEE). Subgroup analyses by implanted stent diameter (≤2.75 versus >2.75 mm) and diabetes were exploratory.

**Results:**

RHI showed only a borderline inverse correlation with in-stent LLL (*ρ* = −0.19, *p* = 0.07) and was not independently associated with it (adjusted *β* = −0.11, 95% CI −0.39 to +0.18, *p* = 0.45); multiple imputation (*β* = −0.08) and GEE (*β* = −0.07) were concordant, and no correlation survived false-discovery-rate correction. Stratification by native reference vessel diameter showed no association, whereas stratification by implanted stent diameter revealed an inverse RHI–LLL correlation confined to small-diameter stents (≤2.75 mm, *n* = 33; *ρ* = −0.375, *p* = 0.032) but not larger stents (*ρ* = −0.055). However, the stent-diameter × RHI interaction was not significant (*p* = 0.48), so this caliber-dependent signal is hypothesis-generating and tracks the achieved implanted caliber rather than the native reference vessel. Inflammation explained neither result. High-sensitivity C-reactive protein (hs-CRP) was unrelated to RHI (*ρ* = −0.02) and to in-stent LLL (*ρ* = +0.15), and adding it to the primary model left the RHI estimate unchanged. Small-caliber recipients had similar hs-CRP but more diabetes (57.6% versus 27.6%, *p* = 0.009) and worse renal function (*p* = 0.008), indicating a higher-risk phenotype rather than a pure caliber stratum.

**Discussion:**

Pre-procedural EndoPAT RHI was not independently associated with in-stent LLL. The exploratory signal confined to small-caliber stents is hypothesis-generating and requires prospective, device-caliber-stratified validation pairing pre- with post-procedural RHI.

## 1 Introduction

Endothelial dysfunction is a central mechanism in atherosclerosis and in the neointimal hyperplasia that follows arterial injury during percutaneous coronary intervention (PCI) (1, 2). Despite the use of drug-eluting stents (DES), in-stent restenosis (ISR) driven by neointimal hyperplasia remains clinically relevant (3–5). The EndoPAT device non-invasively quantifies peripheral microvascular function through the reactive hyperemia index (RHI), a validated surrogate of systemic endothelial function that reflects nitric-oxide-dependent vasodilation and predicts cardiovascular events (6–10).

Two studies using RHI have linked peripheral endothelial function to restenosis, but both localized the predictive signal to the post-procedural rather than the baseline measurement: Komura et al. reported that follow-up RHI independently predicted binary in-stent restenosis in 249 patients (odds ratio 0.13; area under the curve 0.67), whereas pre-procedural RHI did not differ between groups (11); Yamamoto et al. similarly found that 8-month, but not early (3-week), RHI was associated with restenosis after primary PCI (12). To our knowledge, no prior study has related pre-procedural EndoPAT RHI to continuous in-stent late lumen loss (LLL) or examined whether such a relationship tracks the achieved device caliber. This question is clinically relevant because small vessels and small implanted stents carry disproportionately higher restenosis risk (4, 5, 13), and endothelial re-coverage of stent struts is a key determinant of healing (2, 14, 15).

We hypothesized that an association between pre-procedure RHI and in-stent LLL, if present, might be most apparent where the treated caliber is small. Accordingly, the present study evaluated (1) the association between RHI and quantitative coronary angiography (QCA) outcomes after PCI, and (2) whether this association differs by caliber, comparing stratification by implanted stent diameter with stratification by angiographic reference vessel diameter.

## 2 Materials and methods

### 2.1 Study design and population

This was a retrospective observational cohort study at Seoul National University Boramae Medical Center (index PCI performed between February 2009 and June 2016, with follow-up angiography through December 2017). Consecutive PCI patients who underwent EndoPAT assessment were enrolled (*n* = 132 with a valid RHI). Follow-up QCA was available in 91 patients (68.9%) (Supplementary Figure S1). Follow-up coronary angiography was obtained through a combination of scheduled protocol surveillance and clinically-driven indications. Complete-case analysis was the primary approach, and multiple imputation was pre-specified as a sensitivity analysis. Reporting follows the STROBE statement (16). The study was approved by the Institutional Review Board of Seoul National University Boramae Medical Center (approval number: 26-2016-176); owing to the retrospective design, the requirement for informed consent was waived. Demographic data, cardiovascular risk factors, and the medications and laboratory results recorded at the time of the EndoPAT assessment — including high-sensitivity C-reactive protein (hs-CRP), HbA1c, lipid profile, serum creatinine, and estimated glomerular filtration rate (eGFR) which was derived using the four-variable Modification of Diet in Renal Disease equation — were retrieved from the electronic medical record.

### 2.2 EndoPAT and reactive hyperemia index

Peripheral endothelial function was assessed with the EndoPAT-2000 device (Itamar Medical, Caesarea, Israel). RHI was computed as the ratio of post- to pre-occlusion fingertip pulse-amplitude during reactive hyperemia, with contralateral-arm correction, per the manufacturer's algorithm. RHI was measured before PCI (pre-procedural RHI), and an RHI <1.67, a threshold chosen with reference to the cut-offs reported in previous EndoPAT studies (1.35–1.73) (6, 11), was used to define endothelial dysfunction.

### 2.3 Quantitative coronary angiography and stent characteristics

Offline QCA yielded three outcomes: acute gain [post-PCI minimal lumen diameter (MLD)−pre-PCI MLD], in-stent LLL (post-PCI MLD−follow-up MLD), and in-segment LLL (post-PCI in-segment MLD−follow-up in-segment MLD). Late lumen loss served as a continuous restenosis endpoint (17). In-stent LLL was the primary outcome; acute gain and in-segment LLL were secondary outcomes. Because RHI is a single patient-level measurement, the patient-level analysis used one lesion per patient: the index lesion, defined as the first lesion treated at the index procedure (in 60 of the 62 patients with more than one treated lesion, all lesions were treated in the same session). The median interval from PCI to follow-up angiography was 9.4 months (IQR 9.0–10.1). Both the implanted stent diameter and the angiographic reference vessel diameter were recorded. Small caliber was defined as ≤2.75 mm. Because implanted stent diameter and reference vessel diameter can diverge, both definitions were examined, with the reference-vessel-diameter definition as a sensitivity analysis. Implanted devices in the follow-up cohort were predominantly second-generation drug-eluting stents (82 of 91, 90%); the remainder comprised a Genous endothelial-progenitor-capture stent (*n* = 3), a paclitaxel-eluting stent (*n* = 2), a drug-coated balloon (*n* = 2), a first-generation sirolimus-eluting stent (*n* = 1), and a polymer-free stent (*n* = 1). Because a drug-coated balloon leaves no permanent scaffold, late lumen loss in the two drug-coated-balloon cases is a treated-segment rather than an in-stent measurement. These cases were retained in the main analysis, and an additional stent-only analysis (excluding them) and a second-generation-DES-only analysis were performed (Supplementary Table S3).

### 2.4 Statistical analysis

In-stent LLL was right-skewed (skewness=4.6, kurtosis=29.4). Therefore, the primary analysis was Spearman rank correlation, complemented by a multivariable linear regression with HC3 robust standard errors. This model adjusted for RHI, age, diabetes, hypertension, number of diseased vessels, and pre-PCI MLD; pre-PCI diameter stenosis was excluded because of collinearity with pre-PCI MLD (r = −0.94). Inference was based on effect sizes (*β* or *ρ*) with 95% confidence intervals. Given the marked skew, two pre-specified robustness checks [±3SD outlier removal and ln(RHI) transformation] verified that the borderline primary association was not driven by extreme values or distributional assumptions. Missing follow-up was addressed by multiple imputation with chained equations (MICE, m = 20), with smoking included in the imputation model (18, 19). Because 41% of followed patients had multiple treated lesions, all 140 follow-up lesions were re-analyzed with generalized estimating equations (GEE) and a random-intercept mixed-effects model, which provides the intraclass correlation (ICC). To assess dependence on the choice of lesion, the primary model was also refitted with each patient's mean and worst (maximum) in-stent LLL across lesions. Subgroup analyses by implanted stent diameter and by diabetes were exploratory, with Benjamini–Hochberg false-discovery-rate correction (q < 0.10). Differential associations were tested formally with interaction terms added to the primary model and a Fisher r-to-z comparison of correlations, since a difference between a significant and a non-significant subgroup is not itself statistically significant (20). *Post-hoc* device-restricted sensitivity analyses (stent-only, excluding drug-coated balloons, and second-generation-DES-only) assessed robustness to device heterogeneity. Analyses used Python 3.11 (statsmodels, scipy, scikit-learn) with a fixed random seed; *α*=0.05 (two-sided). Precision was quantified *a priori* in terms of the minimum detectable effect. With 91 patients, the study had 80% power (*α*=0.05, two-sided) to detect a Spearman |*ρ*| of 0.29 or greater, and within the 33-patient small-caliber subgroup only 0.47 or greater. To assess whether inflammation, glycaemia, renal function, neurohormonal blockade or smoking confounded the RHI–LLL relationship, ln-transformed hs-CRP, HbA1c, eGFR, *β*-blocker use, angiotensin-converting-enzyme-inhibitor or angiotensin-receptor-blocker use, and smoking status were each added to the primary model. Because these variables differed in availability, every model was compared with the identical model fitted in exactly the same patients (Supplementary Table S4). Baseline characteristics were also compared between recipients of small (≤2.75 mm) and larger stents using Mann–Whitney U tests for continuous variables and chi-square or Fisher exact tests for categorical variables (Supplementary Table S1).

## 3 Results

### 3.1 Study population and QCA distribution

A total of 132 patients were included (mean age 64.1 ± 9.6 years; 72.0% male; 42.4% diabetes; 62.1% hypertension; 64.4% current or former smokers; Table 1). Secondary-prevention therapy was near-universal (statin 97.0%, *β*-blocker 83.3%, angiotensin-converting-enzyme inhibitor or angiotensin-receptor blocker 86.4%). Median hs-CRP was 0.14 mg/dL (IQR 0.05–0.32; available in 93 patients), median eGFR was 79.6 mL/min/1.73 m^2^ (IQR 67.9–91.5), 12.1% had an eGFR below 60 mL/min/1.73 m^2^, and mean HbA1c was 6.5 ± 1.1%. Mean RHI was 1.636 ± 0.466 (range 0.38–3.23), and 84 patients (63.6%) had an RHI below 1.67. Mean implanted stent diameter was 3.06 ± 0.45 mm, and 36% of the follow-up cohort received a small (≤2.75 mm) stent (Table 1). Median in-stent LLL was 0.12 mm (IQR 0.045–0.385) with positive skewness, and acute gain was approximately normally distributed (Table 2). Patients without follow-up angiography were more often current or former smokers than those with follow-up (80.5% versus 57.1%, *p* = 0.017), whereas age, sex (67.0% versus 82.9% male, *p* = 0.10), diabetes, hypertension, RHI and hs-CRP did not differ significantly (Table 1).

**Table 1:** Baseline characteristics of the study population.

Variable	All (*n*=132)	F/U QCA (*n*=91)	No F/U QCA (*n*=41)	p
*Demographics*				
Age (years)	64.1±9.6	63.9±9.7	64.7±9.5	0.88
Male sex	95 (72.0%)	61 (67.0%)	34 (82.9%)	0.10
Body mass index (kg/m²)	24.3±3.2	24.3±2.7	24.4±4.0	0.64
*Cardiovascular risk factors*				
Diabetes mellitus	56 (42.4%)	35 (38.5%)	21 (51.2%)	0.24
Hypertension	82 (62.1%)	57 (62.6%)	25 (61.0%)	0.99
Current or former smoker	85 (64.4%)	52 (57.1%)	33 (80.5%)	0.017
Prior cerebrovascular disease	14 (10.6%)	9 (9.9%)	5 (12.2%)	0.76
Peripheral artery disease	17 (12.9%)	12 (13.2%)	5 (12.2%)	0.90
*Laboratory*				
hs-CRP (mg/dL), *n*=93	0.14 [0.05–0.32]	0.08 [0.05–0.23]	0.23 [0.05–0.65]	0.08
HbA1c (%), *n*=110	6.5±1.1	6.4±1.0	6.8±1.3	0.19
LDL cholesterol (mg/dL), *n*=127	78±28	76±24	82±35	0.65
HDL cholesterol (mg/dL), *n*=127	44±11	43±10	45±13	0.46
Triglycerides (mg/dL), *n*=126	105 [78–152]	105 [80–152]	109 [74–152]	0.88
Creatinine (mg/dL), *n*=124	0.90 [0.77–1.04]	0.87 [0.77–1.04]	0.95 [0.81–1.05]	0.28
eGFR (mL/min/1.73 m²), *n*=124	79.6 [67.9–91.5]	79.6 [67.9–93.2]	80.2 [68.6–85.8]	0.59
eGFR <60 mL/min/1.73 m²	15 (12.1%)	11 (12.6%)	4 (10.8%)	1.00
*Medication at the time of EndoPAT*				
Statin	128 (97.0%)	89 (97.8%)	39 (95.1%)	0.59
Beta-blocker	110 (83.3%)	76 (83.5%)	34 (82.9%)	0.87
ACEi or ARB	114 (86.4%)	80 (87.9%)	34 (82.9%)	0.62
Calcium-channel blocker	42 (31.8%)	30 (33.0%)	12 (29.3%)	0.83
*Vascular function*				
RHI	1.636±0.466	1.639±0.445	1.629±0.514	0.60
RHI <1.67	84 (63.6%)	56 (61.5%)	28 (68.3%)	0.58
baPWV, right (cm/s), *n*=99	1,568±280	1,588±289	1,517±256	0.46
ABI, right, *n*=98	1.12±0.12	1.13±0.12	1.11±0.11	0.11
*Angiographic and procedural*				
No. of diseased vessels	2 [1–3]	2 [2–3]	2 [1–3]	0.83
Target (index) lesion in LAD	71 (53.8%)	52 (57.1%)	19 (46.3%)	0.34
Reference vessel diameter (mm), *n*=124	3.12±0.58	3.09±0.53	3.18±0.67	0.32
Pre-PCI MLD (mm), *n*=131	0.85±0.63	0.84±0.62	0.85±0.65	0.91
Lesion length (mm), *n*=124	18.2±11.8	19.3±12.3	15.7±10.5	0.11
Implanted stent diameter (mm), *n*=131	3.06±0.45	3.05±0.45	3.11±0.46	0.35
Small stent ≤2.75 mm	43/131 (32.8%)	33/91 (36.3%)	10/40 (25.0%)	0.29
Implanted stent length (mm), *n*=131	24.5±7.6	25.1±7.5	22.9±7.6	0.12
Second-generation DES	120 (90.9%)	82 (90.1%)	38 (92.7%)	0.75
Other device*	12 (9.1%)	9 (9.9%)	3 (7.3%)	0.75
Follow-up interval (months)	—	9.4 [9.0–10.1]	—	—

Values are mean±SD, *n* (%), or median [IQR]. *Other devices: Genous endothelial-progenitor-capture stent, paclitaxel-eluting stent, drug-coated balloon, first-generation sirolimus-eluting stent, polymer-free stent, and plain balloon angioplasty. Laboratory values were obtained at the time of the EndoPAT assessment. *p* values compare patients with and without follow-up QCA (Mann–Whitney U or chi-square/Fisher exact test). hs-CRP, high-sensitivity C-reactive protein; eGFR, estimated glomerular filtration rate; ACEi, angiotensin-converting-enzyme inhibitor; ARB, angiotensin-receptor blocker; baPWV, brachial–ankle pulse wave velocity; ABI, ankle–brachial index; F/U, follow-up; RHI, reactive hyperemia index; LAD, left anterior descending; DES, drug-eluting stent.

**Table 2:** Distribution of QCA outcomes (follow-up QCA subset unless noted).

Variable	N	Mean±SD	Median [IQR]	Skewness
Acute gain (mm)	131	1.679±0.696	1.67 [1.22–2.19]	−0.24
In-stent LLL (mm)	91	0.291±0.534	0.12 [0.045–0.385]	4.61
In-segment LLL (mm)	91	0.149±0.540	0.08 [−0.10–0.23]	4.67

Acute gain was available in 131 patients. Extreme non-normality of LLL prompted the use of Spearman and HC3-robust methods. For the two drug-coated-balloon cases, late lumen loss is a treated-segment measurement. LLL, late lumen loss.

### 3.2 RHI and QCA outcomes

RHI showed a borderline inverse correlation with in-stent LLL (*ρ*=−0.19, 95%CI −0.38 to +0.02, *p* = 0.07; Figure 1A) and a nominal inverse correlation with in-segment LLL (*ρ*=−0.21, *p* = 0.044; Figure 1B); neither survived multiplicity correction across the three QCA outcomes (BH-FDR q = 0.11; Table 3). There was no association with acute gain (*ρ*=+0.01, *p* = 0.95; Figure 1C). Median in-stent LLL decreased monotonically across RHI tertiles (0.190, 0.145 and 0.100 mm from the lowest to the highest tertile). In the primary multivariable model, RHI was not independently associated with in-stent LLL (*β*=−0.11, 95%CI −0.39 to +0.18, *p* = 0.45; adjusted R^2^ = 0.02; Table 4), and no covariate reached significance. This null result was robust: multiple imputation for the 31% missing follow-up was concordant with the complete-case estimate (*β*=−0.08, 95%CI −0.33 to +0.18, *p* = 0.57), as were ±3SD outlier removal (*ρ*=−0.19, *p* = 0.08) and ln(RHI) transformation (*ρ*=−0.19, *p* = 0.07). Excluding the two drug-coated-balloon cases gave similar results (*ρ*=−0.18, *p* = 0.09; adjusted *β*=−0.10, *p* = 0.48; Supplementary Table S3).

**Figure 1:**
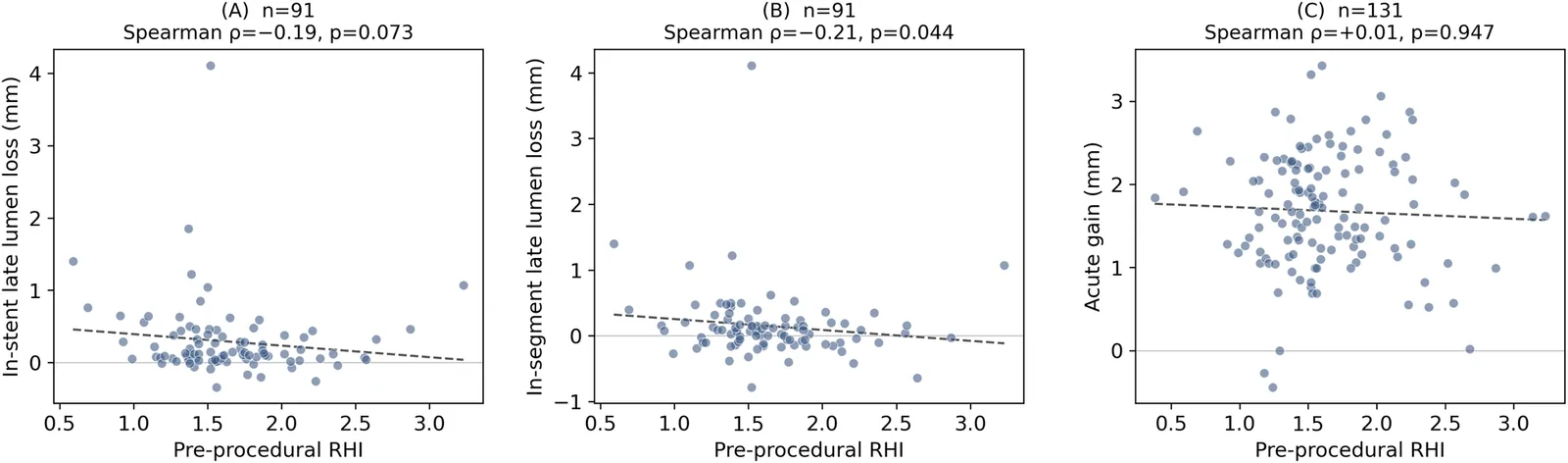
RHI versus quantitative coronary angiographic outcomes. **(A)** In-stent late lumen loss (*n* = 91; Spearman *ρ*=−0.19, *p* = 0.07); **(B)** in-segment late lumen loss (*n* = 91; *ρ*=−0.21, *p* = 0.044, not significant after Benjamini–Hochberg correction, q = 0.11); **(C)** acute gain (*n* = 131; *ρ*=+0.01, *p* = 0.95). Dashed lines, linear trend; *x*-axis, pre-procedural RHI.

**Table 3:** Spearman correlations between RHI and QCA outcomes.

Pair	N	*ρ*	95% CI	p
*Pre-specified QCA correlations*				
RHI – acute gain	131	+0.006	−0.166 to +0.177	0.95
RHI – in-stent LLL	91	−0.19	−0.38 to +0.02	0.07
RHI – in-segment LLL	91	−0.21	−0.40 to −0.01	0.044*
*Exploratory subgroup correlations (RHI* vs. *in-stent LLL)*				
DM+: RHI – LLL	35	−0.366	−0.62 to −0.04	0.030*
DM−: RHI – LLL	56	−0.020	−0.28 to +0.24	0.88
Small stent ≤2.75 mm	33	−0.375	−0.64 to −0.04	0.032*
Larger stent >2.75 mm	58	−0.055	−0.31 to +0.21	0.68

* *p*<0.05 (nominal). Across the three pre-specified QCA correlations (acute gain, in-stent LLL, in-segment LLL), Benjamini–Hochberg FDR q=0.11 for both in-stent and in-segment LLL (neither significant after correction). Exploratory rows are RHI–in-stent LLL correlations within the indicated subgroup. DM, diabetes mellitus.

**Table 4:** Primary multivariable model for in-stent LLL (*n*=91; R²=0.086, adjusted R²=0.021; HC3 robust SE).

Variable	*β*	SE (HC3)	95% CI	p
RHI	−0.109	0.145	−0.394 to +0.176	0.45
Age	+0.003	0.005	−0.006 to +0.012	0.50
Diabetes mellitus	+0.239	0.143	−0.041 to +0.519	0.09
Hypertension	+0.049	0.102	−0.151 to +0.248	0.63
No. diseased vessels	−0.113	0.071	−0.251 to +0.025	0.11
Pre-PCI MLD	−0.092	0.060	−0.210 to +0.026	0.13

Pre-PCI diameter stenosis was excluded for collinearity with pre-PCI MLD (r=−0.94). Multiple imputation (m=20) gave a concordant RHI estimate (β=−0.08, 95%CI −0.33 to +0.18, *p*=0.57). MLD, minimal lumen diameter.

### 3.3 Exploratory subgroup analyses by stent caliber and diabetes

The exploratory subgroup finding depended on which caliber metric defined it. Stratification by native reference vessel diameter showed no association at any cut-off (≤2.75 mm: *ρ*=+0.03, *p* = 0.92, *n* = 20; ≤2.5 mm: *ρ*=−0.28, *p* = 0.37; reference diameter × RHI interaction *p* = 0.68). By contrast, stratification by implanted stent diameter revealed an inverse RHI–in-stent LLL correlation confined to small-diameter stents (Table 5; Figure 2): ≤2.75 mm (*n* = 33), *ρ*=−0.375, 95%CI −0.636 to −0.038, *p* = 0.032, BH-FDR q = 0.06, versus *ρ*=−0.055 (*p* = 0.68, *n* = 58) for larger stents. In *post hoc*, exploratory threshold analyses of nested subsets, the correlation was numerically stronger at narrower cut-offs (*ρ*=−0.20 at ≤3.5 mm to −0.42 at ≤2.5 mm; Supplementary Figure S2) and was mirrored by a split on achieved post-PCI MLD (smaller half: *ρ*=−0.31, *p* = 0.035). Because the formal stent-diameter × RHI interaction was not statistically significant (*β*=−0.24, *p* = 0.48; Fisher r-to-z *p* = 0.13), this caliber-dependent pattern is hypothesis-generating rather than a demonstrated effect modification; comparable RHI distributions between small- and large-stent groups (medians 1.52 vs 1.56, *p* = 0.74) argue against a measurement-bias explanation. In a diabetes-stratified analysis, the RHI–in-stent LLL correlation was stronger in patients with diabetes (*ρ*=−0.366, *p* = 0.030, *n* = 35; Supplementary Figure S3) than without (*ρ*=−0.020, *p* = 0.88, *n* = 56). The diabetes × RHI interaction was nominally significant in the primary covariate model (*β*=−0.58, *p* = 0.018) but not after additional adjustment for lesion length and smoking (*p* = 0.35; *n* = 82), and it was not corrected for multiplicity. Small and large caliber stent recipients differed in baseline phenotype rather than in inflammation (Supplementary Table S1). hs-CRP was virtually identical (0.085 versus 0.080 mg/dL, *p* = 0.70), whereas the small-caliber stent group had more diabetes (57.6% versus 27.6%, *p* = 0.009), a lower eGFR (71.6 versus 83.1 mL/min/1.73 m^2^, *p* = 0.008) and more diseased vessels (*p* = 0.009), and received *β*-blockers (69.7% versus 91.4%, *p* = 0.017) and angiotensin-converting-enzyme inhibitors or angiotensin-receptor blockers (78.8% versus 93.1%, *p* = 0.09) less often. Nineteen of the 33 small-caliber patients had diabetes, so the caliber and diabetes subgroups overlapped substantially and cannot be regarded as independent signals.

**Table 5:** Small (≤2.75 mm) versus larger (>2.75 mm) implanted stent diameter [exploratory].

Variable	Small ≤2.75 mm (*n*=33)	Larger >2.75 mm (*n*=58)	p
*Group comparison — median [IQR], Mann–Whitney U*			
RHI	1.520 [1.37–1.87]	1.560 [1.39–1.81]	0.74
Acute gain (mm)	1.380 [1.06–1.91]	1.825 [1.37–2.28]	0.014*
In-stent LLL (mm)	0.150 [0.08–0.46]	0.120 [0.03–0.34]	0.21
*Within-group RHI–in-stent LLL correlation*			
Spearman ρ	−0.375	−0.055	—
*p* value	0.032*	0.68	—

Median [IQR]; Mann–Whitney U test. *p<0.05. The stent-diameter×RHI interaction was not significant (β=−0.24, *p*=0.48; Fisher r-to-z *p*=0.13). Sensitivity by reference vessel diameter did not reproduce the association at any cut-off (≤2.75 mm: ρ=+0.03, *p*=0.92, *n*=20); RHI distributions were comparable between groups (*p*=0.74). A full baseline comparison of the two caliber groups is provided in Supplementary Table S1.

**Figure 2:**
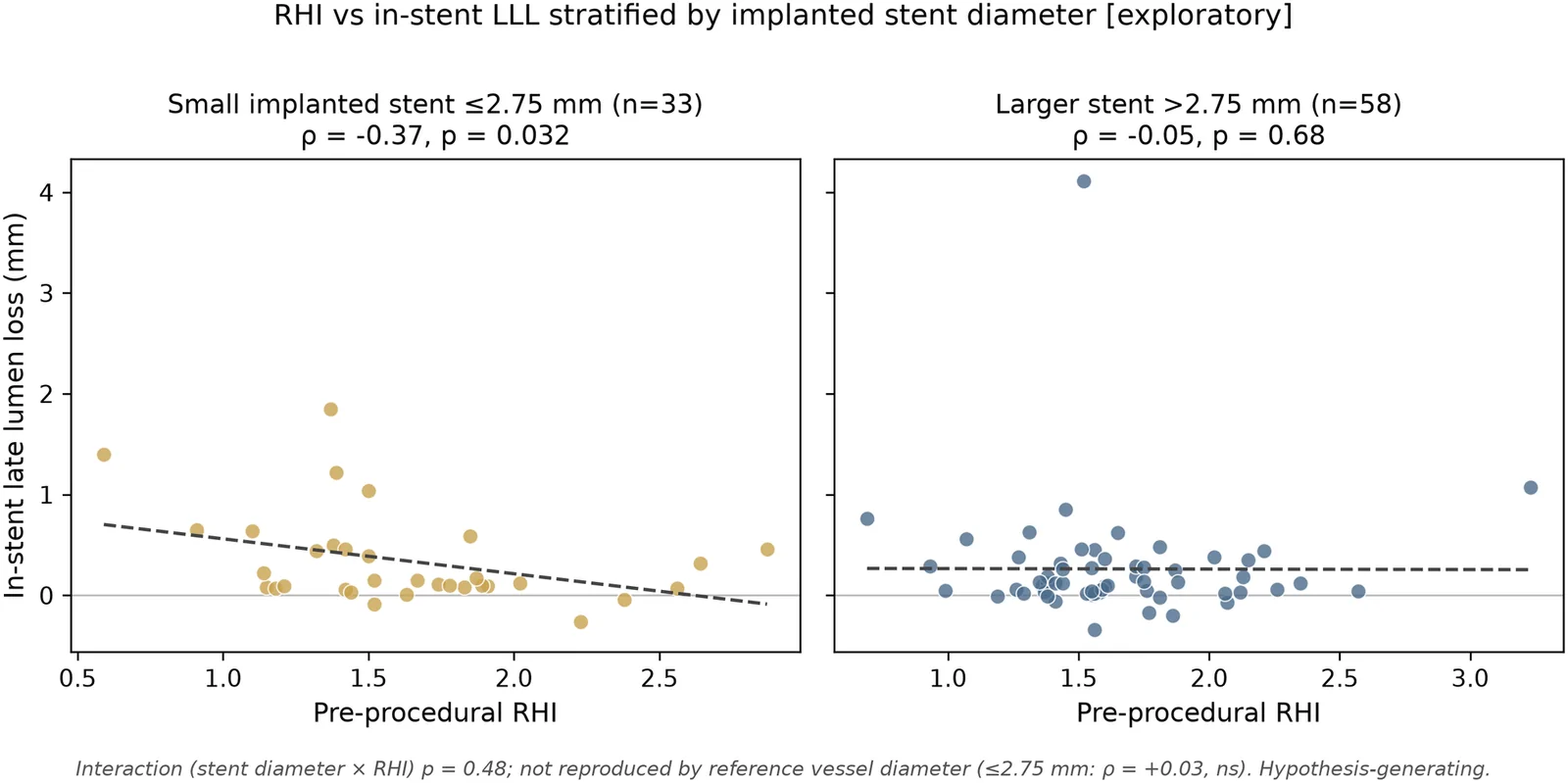
RHI versus in-stent LLL stratified by implanted stent diameter. Left, small stent ≤2.75 mm (*n* = 33; *ρ*=−0.375, *p* = 0.032); right, larger stent >2.75 mm (*n* = 58; *ρ*=−0.055, *p* = 0.68). The stent-diameter × RHI interaction was not significant (*p* = 0.48) and the association was not reproduced by reference vessel diameter (≤2.75 mm: *ρ*=+0.03, ns).

### 3.4 Competing predictors and lesion-level clustering

Lesion length was associated with in-stent LLL (*ρ*=+0.27, *p* = 0.015) but was independent of RHI (*ρ*=+0.03, *p* = 0.79); adding lesion length to the primary model left the RHI estimate essentially unchanged (*β* −0.11 to −0.08), indicating a competing predictor rather than a confounder.

In the lesion-level analysis of all 140 follow-up lesions, within-patient clustering was modest (ICC=0.11); a GEE model reproduced the non-significant inverse association (*β*=−0.07, 95%CI −0.25 to +0.11, *p* = 0.46) and a random-intercept model was concordant (Supplementary Table S2), confirming robustness to the analytic unit. The adjusted estimate was also insensitive to the choice of lesion: the RHI coefficient was −0.09 (*p* = 0.54) for each patient's mean in-stent LLL and −0.05 (*p* = 0.71) for the worst lesion (Supplementary Table S2), with unadjusted correlations similar to that of the index lesion (*ρ*=−0.22, *p* = 0.038 and *ρ*=−0.21, *p* = 0.049, respectively).

### 3.5 Inflammation, metabolic status, and neurohormonal blockade

hs-CRP was available in 93 patients overall and in 63 of the 91 with follow-up angiography (median 0.14 mg/dL, IQR 0.05–0.32). It was not correlated with RHI (*ρ*=−0.024, 95%CI −0.23 to +0.18, *p* = 0.82), with in-stent LLL (*ρ*=+0.148, −0.10 to +0.38, *p* = 0.25), with in-segment LLL (*ρ*=+0.035, *p* = 0.79), or with implanted stent diameter (*ρ*=+0.019, *p* = 0.88). In the 63 patients with hs-CRP available, the primary model yielded an RHI coefficient of −0.239 (95%CI −0.513 to +0.035, *p* = 0.09); adding ln(hs-CRP) changed it to −0.237 (−0.508 to +0.034), and ln(hs-CRP) itself was not associated with in-stent LLL (*β*=−0.023, *p* = 0.62). The same pattern held for eGFR (RHI *β* −0.100 to −0.108 in 87 patients), HbA1c (−0.220 to −0.244 in 73 patients), *β*-blocker use (−0.109 to −0.111) and angiotensin-converting-enzyme-inhibitor or angiotensin-receptor-blocker use (−0.109 to −0.109), each compared within identical patients (Supplementary Table S4). Smoking status produced the largest change, attenuating the RHI coefficient from −0.109 to −0.068, but was not itself associated with in-stent LLL (*β*=+0.174, *p* = 0.20). However, the RHI coefficient was larger in the subsets with complete laboratory data than in the whole follow-up cohort (−0.239 in the 63 patients with hs-CRP and −0.220, *p* = 0.04, in the 73 with HbA1c, versus −0.109 in all 91), so these subsets are not exchangeable with the full cohort. The covariate comparisons above are therefore valid within each subset and are not intended to revise the primary estimate. Neither RHI nor in-stent LLL differed by *β*-blocker or angiotensin-converting-enzyme-inhibitor/angiotensin-receptor-blocker use (all *p* ≥ 0.58).

## 4 Discussion

In this retrospective cohort of 132 Korean patients undergoing PCI, peripheral microvascular function (RHI) was not independently associated with in-stent late lumen loss. The unadjusted inverse correlation was borderline and non-significant (*ρ*=−0.19, *p* = 0.07), the adjusted estimate was null (*β*=−0.11, *p* = 0.45). This conclusion was also stable across two pre-specified robustness analyses: multiple imputation for the 31% missing follow-up (*β*=−0.08, *p* = 0.57) and lesion-level clustering-aware models (GEE *β*=−0.07; ICC=0.11). In an exploratory, caliber-stratified analysis, lower RHI was associated with greater in-stent LLL among patients who received small-diameter stents (≤2.75 mm; *ρ*=−0.375, *p* = 0.032), but not among those with larger stents.

Our findings both extend and qualify the prior literature. In the two peripheral-arterial-tonometry studies of restenosis, Komura et al. and Yamamoto et al. both localized the predictive signal to the post-procedural measurement: follow-up RHI predicted binary in-stent restenosis, whereas baseline or early RHI did not differ between groups (11, 12). Two features distinguish the present study. First, we examined pre-procedural RHI. Second, we used continuous QCA measurements rather than a dichotomous endpoint (such as ISR). Pre-procedural RHI did not independently predict LLL, consistent with the non-predictive baseline measurements in these prior tonometry studies (11, 12), but the follow-up measurement has instead been the informative one (11, 12, 21). This pattern suggests that endothelial function measured after the vascular injury and healing after PCI may carry more restenosis-relevant information than a single pre-procedural measurement, consistent with restenosis being driven by peri-procedural injury and repair rather than by baseline systemic endothelial function alone (2). Our null baseline association therefore does not contradict prior work; it delimits the prognostic window in which EndoPAT is informative.

The inverse correlation between RHI and in-stent LLL emerged with implanted stent diameter but not with reference vessel diameter. Because the between-group difference was not significant (interaction *p* = 0.48; Fisher r-to-z *p* = 0.13), this subgroup association is exploratory. We therefore interpret it in light of established evidence on small-caliber restenosis rather than mechanistic speculation.

Several established observations make an endothelial signal most detectable in small implanted stents. Endothelial shear stress has been linked to the post-stenting neointimal response, although its precise role in human in-stent restenosis remains debated (22, 23). The nitric-oxide pathway offers more direct evidence. Restoring endothelial nitric-oxide synthase inhibits neointimal formation after arterial injury, and nitric oxide suppresses vascular smooth-muscle proliferation (24–26). Impaired systemic endothelial function reflects reduced nitric-oxide bioavailability and therefore acts on this same pathway. Neointimal hyperplasia is, moreover, the principal source of lumen loss in small stents (4, 27); where it dominates, an endothelial signal is least diluted by competing processes.

The absence of a signal for reference vessel diameter need not invoke additional biology. The endothelium heals the implanted stent lumen, not the pre-procedural reference segment (2, 14, 15), so the achieved caliber is the relevant substrate. Two further factors weaken the reference-diameter stratification. First, reference vessel diameter is interpolated from adjacent segments and is more measurement-variable than a discrete, operator-verified stent diameter; such non-differential error attenuates true effect modification toward the null (regression dilution) (28), an effect compounded by the smaller subgroup (*n* = 20 versus 33). Second, implanted stent diameter is itself a post-procedural, operator-chosen variable, so this subgroup is not a baseline stratum. This pattern mirrors the wider restenosis literature, in which the achieved minimal stent area on intravascular ultrasound, rather than the reference vessel size, independently predicts in-stent restenosis (29). Clinically, the signal therefore tracks the achieved procedural result rather than native anatomy. A small implanted stent is not a diagnosis of small-vessel disease.

Two mechanisms that could plausibly modulate microvascular recovery after stent implantation — systemic inflammation and neurohormonal activation — merit specific comment. Vascular inflammation is closely coupled to re-endothelialisation and repair after stenting (2), and experimental work implicates angiotensin II in smooth-muscle-cell proliferation and myointimal thickening after arterial injury, an effect attenuated by angiotensin-converting-enzyme inhibition (30, 31). In this cohort, the available inflammatory marker did not behave as a confounder. hs-CRP was unrelated to RHI, to in-stent LLL and to implanted stent diameter, and its inclusion left the RHI coefficient essentially unchanged.

Neurohormonal blockade was too uniform to be informative. More than four in five patients were taking a *β*-blocker and nearly nine in ten an angiotensin-converting-enzyme inhibitor or angiotensin-receptor blocker, so residual between-patient variation in neurohormonal activation could not be modelled, and no direct neurohormonal biomarker — for example N-terminal pro-B-type natriuretic peptide, catecholamines, or renin–angiotensin–aldosterone measures — was recorded. These analyses therefore argue against systemic inflammation, as indexed by a single pre-procedural hs-CRP measurement, as a confounder of the present findings, but they do not exclude an inflammatory or neurohormonal contribution to in-stent late lumen loss, which a prospective study with serial biomarker sampling would be far better placed to test.

The mechanistic account given above is not the only possible reading of the caliber-dependent signal. The comparison of small-caliber stent with large-caliber stent recipients qualifies it further. The two groups were indistinguishable in hs-CRP, yet the small-caliber group carried a heavier metabolic and renal burden — a diabetes prevalence of 58% versus 28%, a lower eGFR and more extensive coronary disease — and received neurohormonal blockade less often. A small implanted stent therefore marks a higher-risk patient phenotype as well as a smaller treated caliber. Also, because 19 of the 33 small-caliber patients also had diabetes, the caliber and diabetes subgroups are not independent; the diabetes × RHI interaction was itself nominally significant in the primary covariate model, although not after further adjustment. This is a further reason, beyond the non-significant stent-diameter × RHI interaction, to treat the caliber-dependent association as hypothesis-generating, and it sharpens the design requirement for any confirmatory study. Pre-stratification by intended device diameter must be accompanied by balance or adjustment for diabetes and renal function.

Several features of this cohort bear on interpretation. This was a single-center Korean series in which EndoPAT was performed selectively, and roughly one-third of treated lesions received small-diameter stents. Our data do not support routine pre-procedural EndoPAT to predict angiographic restenosis in unselected patients. If the small-stent signal is pursued, it should be tested prospectively in an adequately powered cohort pre-stratified by intended device diameter. Ideally, such a study would pair baseline with post-procedural RHI, so that the more informative post-injury measurement can be evaluated alongside the baseline value.

As expected for patients referred for PCI, the cohort was shifted toward endothelial dysfunction, with 63.6% having an RHI below 1.67. Such restriction of range can distort an exposure–outcome association in either direction, attenuating a correlation when the underlying relationship is linear but accentuating one when the sampled range covers a steeper segment. The measured range nevertheless remained wide (RHI 0.38–3.23; SD 0.466), and the adjusted estimate is a regression slope, which selection on the exposure does not bias, so restriction of range is unlikely to explain the borderline primary result.

### 4.1 Limitations

First, the retrospective single-center design and selective EndoPAT referral introduce selection bias. Because follow-up angiography was obtained partly on clinical indication rather than by protocol alone, the followed subset may be enriched for recurrent symptoms or restenosis, potentially biasing late-lumen-loss estimates and the exposure–outcome association. Second, follow-up angiography was missing in 31.1% of patients; missingness was related to smoking status, indicating that the data were not missing completely at random, and although the pre-specified multiple-imputation analysis was concordant with the complete-case result, residual bias under a not-at-random mechanism cannot be excluded. Third, the modest sample size is a potential limitation that likely contributed to the borderline primary result. With 91 patients, the study had 80% power (*α*=0.05, two-sided) to detect a Spearman |*ρ*| of only 0.29 or greater, and within the 33-patient small-caliber subgroup only 0.47 or greater. Accordingly, the borderline primary result should be read as “not established” rather than “no effect,” since the 95% confidence interval for the primary correlation (−0.38 to +0.02) remains compatible with a small-to-moderate inverse association. For the same reason, the nominally significant small-caliber correlation may equally be an overestimate arising from low power. Fourth, all subgroup and sensitivity analyses beyond the pre-specified plan are exploratory; the stent-diameter difference was not statistically significant, was not reproduced by reference vessel diameter, and rests on a post-procedural, operator-chosen stratifier (so it is not a baseline stratum and residual selection cannot be excluded). Fifth, the design is observational; although RHI temporally preceded the angiographic outcome, causation cannot be inferred. Sixth, inflammatory and neurohormonal characterisation was limited. hs-CRP was a single pre-procedural measurement available in 63 of the 91 followed patients. Its distribution was low in this electively treated cohort, and no neurohormonal biomarker was recorded. The absence of confounding by these variables should therefore be read as the absence of a detectable signal rather than as evidence of no effect.

### 4.2 Conclusion

In this retrospective cohort, pre-procedural peripheral endothelial function measured by EndoPAT was not independently associated with in-stent late lumen loss after PCI—a null finding robust to multiple imputation and to lesion-level, clustering-aware analysis. A one-time, pre-procedural RHI is therefore unlikely to serve as a stand-alone predictor of angiographic restenosis in unselected patients. An exploratory signal was confined to small-caliber PCI. Among recipients of small-diameter stents (≤2.75 mm), lower pre-procedural RHI was associated with greater in-stent LLL, and this signal tracked the achieved implanted caliber rather than the native reference vessel. Because the stent-diameter × RHI interaction was not statistically significant and was not reproduced by reference vessel diameter, the observation is hypothesis-generating rather than confirmatory; it is of interest chiefly because in-stent restenosis remains clinically relevant in small-caliber lesions even in the drug-eluting-stent era.

These data do not support adopting pre-procedural EndoPAT for restenosis risk stratification in general PCI. To determine whether this caliber-dependent signal is real and clinically actionable, future prospective studies are required. These studies should be pre-stratified by intended device diameter and pair pre- with post-procedural RHI. Furthermore, investigators must adjust for diabetes and renal function while including serial inflammatory and neurohormonal markers.

## Data Availability

The original contributions presented in the study are included in the article/Supplementary Material, further inquiries can be directed to the corresponding author.
