# Supplementary material for: Peripheral microvascular function and in-stent late lumen loss after percutaneous coronary intervention: a retrospective cohort study with an exploratory analysis by implanted stent diameter
Source: Front Cardiovasc Med. 2026 Oct 27;13:1940897. doi: 10.3389/fcvm.2026.1940897 (PMC13748259; doi:10.3389/fcvm.2026.1940897)
Supplement: Supplementary file 1 [file Datasheet1.pdf]

Supplementary Material

Supplementary Table 1. Baseline characteristics, laboratory values, and medications according to implanted stent diameter (follow-up QCA cohort, n=91).

Variable	Small stent ≤2.75 mm (n=33)	Larger stent >2.75 mm (n=58)	p
Inflammation			
hs-CRP (mg/dL)	0.085 [0.050–0.250] (n=26)	0.080 [0.040–0.220] (n=37)	0.70
Metabolic and renal			
Diabetes mellitus	19/33 (57.6%)	16/58 (27.6%)	0.009
HbA1c (%)	6.3 [5.7–7.0] (n=28)	6.1 [5.8–6.7] (n=45)	0.58
eGFR (mL/min/1.73 m²)	71.6 [60.4–85.3] (n=31)	83.1 [72.4–93.7] (n=56)	0.008
LDL cholesterol (mg/dL)	75 [62–90] (n=31)	70 [61–91] (n=57)	0.71
HDL cholesterol (mg/dL)	44 [38–50] (n=31)	41 [35–47] (n=57)	0.24
Triglycerides (mg/dL)	87 [76–121] (n=31)	118 [84–152] (n=56)	0.069
Other risk factors			
Age (years)	68 [59–73]	64 [57–69]	0.13
Male sex	18/33 (54.5%)	43/58 (74.1%)	0.093
Hypertension	25/33 (75.8%)	32/58 (55.2%)	0.084
Current or former smoker	17/33 (51.5%)	35/58 (60.3%)	0.55
Body mass index (kg/m²)	23.4 [22.0–25.1]	24.4 [22.5–26.3]	0.36
Neurohormonal and other therapy			
Beta-blocker	23/33 (69.7%)	53/58 (91.4%)	0.017
ACEi or ARB	26/33 (78.8%)	54/58 (93.1%)	0.090
Statin	33/33 (100%)	56/58 (96.6%)	0.53
Calcium-channel blocker	15/33 (45.5%)	15/58 (25.9%)	0.093
Vascular function			
RHI	1.520 [1.370–1.870]	1.560 [1.387–1.810]	0.74
baPWV, right (cm/s)	1500 [1442–1720] (n=23)	1547 [1355–1754] (n=48)	0.59
ABI, right	1.14 [0.98–1.18] (n=22)	1.14 [1.11–1.21] (n=48)	0.25
Angiographic			
No. of diseased vessels	2 [2–3]	2 [1–3]	0.009
Target (index) lesion in LAD	20/33 (60.6%)	32/58 (55.2%)	0.78
Reference vessel diameter (mm)	2.78 [2.45–3.01] (n=30)	3.12 [2.93–3.50] (n=55)	<0.001
Pre-PCI MLD (mm)	0.71 [0.30–1.00]	0.89 [0.49–1.33]	0.11
Lesion length (mm)	19.1 [9.7–31.5] (n=30)	15.4 [10.7–20.7] (n=55)	0.19
Implanted stent length (mm)	28.0 [22.0–33.0]	23.5 [18.0–28.0]	0.060
QCA outcomes			
Acute gain (mm)	1.38 [1.06–1.91]	1.83 [1.36–2.28]	0.014
In-stent late lumen loss (mm)	0.150 [0.080–0.460]	0.120 [0.030–0.343]	0.21
In-segment late lumen loss (mm)	0.040 [−0.060–0.160]	0.090 [−0.097–0.240]	0.59

Values are median [IQR] or n/N (%). Continuous variables were compared with the Mann–Whitney U test and categorical variables with the chi-square test or Fisher exact test where expected counts were <5. Denominators are shown where data were incomplete. These comparisons are descriptive and were not corrected for multiple testing, so individual p-values should be interpreted accordingly. The difference in reference vessel diameter is expected, because stent size is chosen from it. Inflammation, as indexed by hs-CRP, did not differ between caliber groups, whereas the small-caliber group had a higher prevalence of diabetes, lower eGFR, more extensive coronary disease, and less frequent neurohormonal blockade. hs-CRP, high-sensitivity C-reactive protein; eGFR, estimated glomerular filtration rate; ACEi, angiotensin-converting-enzyme inhibitor; ARB, angiotensin-receptor blocker; baPWV, brachial–ankle pulse wave velocity; ABI, ankle–brachial index; RHI, reactive hyperemia index; LAD, left anterior descending; MLD, minimal lumen diameter; QCA, quantitative coronary angiography.

Supplementary Table 2. Adjusted RHI association across analytic strategies (lesion-level clustering and lesion-selection sensitivity analyses).

Outcome / strategy	Unit	N	Adjusted β	95% CI	p
In-stent LLL — patient-level OLS	Patient	91	−0.109	−0.394 to +0.176	0.45
In-stent LLL — patient mean of all lesions	Patient	91	−0.087	−0.361 to +0.188	0.54
In-stent LLL — worst (maximum) lesion	Patient	91	−0.053	−0.338 to +0.231	0.71
In-stent LLL — GEE (α=0.10)	Lesion	140	−0.067	−0.245 to +0.111	0.46
In-stent LLL — random-intercept (ICC=0.11)	Lesion	140	−0.068	−0.272 to +0.137	0.52
In-segment LLL — GEE	Lesion	140	−0.048	−0.209 to +0.114	0.56
Acute gain — GEE	Lesion	199	−0.055	−0.199 to +0.089	0.45

Covariates: age, diabetes, hypertension, number of diseased vessels, pre-PCI MLD. GEE, generalized estimating equations; ICC, intraclass correlation; OLS, ordinary least squares.

Supplementary Table 3. Device-restricted sensitivity analyses of the RHI–in-stent late lumen loss correlation.

Analysis	Devices	N	ρ	p
Primary (all devices)	all	91	−0.19	0.073
Primary, stent-only	exclude DCB	89	−0.18	0.092
Primary, 2nd-generation DES only	2G-DES	82	−0.13	0.230
Small stent ≤2.75 mm (all)	all	33	−0.375	0.032
Small stent ≤2.75 mm, stent-only	exclude DCB	32	−0.351	0.049
Small stent ≤2.75 mm, 2G-DES only	2G-DES	29	−0.291	0.125

Spearman correlations between RHI and in-stent late lumen loss. Stent-only excludes the two drug-coated-balloon cases (no permanent scaffold), in which late lumen loss is a treated-segment rather than an in-stent measurement; in the stent-only set, the adjusted RHI coefficient was −0.100 (95% CI −0.381 to +0.181, p=0.48). The small-stent signal attenuated under the second-generation-DES-only restriction, consistent with the reduced sample size, reinforcing its exploratory status. DCB, drug-coated balloon; DES, drug-eluting stent; 2G-DES, second-generation drug-eluting stent.

Supplementary Table 4. Covariate-adjusted sensitivity models for the association between RHI and in-stent late lumen loss.

Model	n	RHI β (95% CI)	p	Added covariate β (95% CI), p
Pre-specified primary model (reference)	91	−0.109 (−0.394 to +0.176)	0.45	—
Inflammation — ln(hs-CRP)				
Same patients, without ln(hs-CRP)	63	−0.239 (−0.513 to +0.035)	0.09	—
Same patients, with ln(hs-CRP)	63	−0.237 (−0.508 to +0.034)	0.09	−0.023 (−0.115 to +0.068), p=0.62
Renal function — eGFR				
Same patients, without eGFR	87	−0.100 (−0.388 to +0.189)	0.50	—
Same patients, with eGFR	87	−0.108 (−0.403 to +0.187)	0.47	+0.002 (−0.003 to +0.008), p=0.37
Glycaemia — HbA1c				
Same patients, without HbA1c	73	−0.220 (−0.431 to −0.009)	0.04†	—
Same patients, with HbA1c	73	−0.244 (−0.479 to −0.010)	0.04†	+0.170 (−0.210 to +0.550), p=0.38
Covariates available in all 91 patients				
Primary model + beta-blocker	91	−0.111 (−0.395 to +0.173)	0.44	−0.068 (−0.384 to +0.248), p=0.67
Primary model + ACEi or ARB	91	−0.109 (−0.401 to +0.182)	0.47	−0.001 (−0.255 to +0.252), p=0.99
Primary model + smoking status	91	−0.068 (−0.351 to +0.214)	0.64	+0.174 (−0.091 to +0.439), p=0.20

All models are linear regressions of in-stent late lumen loss with HC3 robust standard errors and the pre-specified covariate set (RHI, age, diabetes, hypertension, number of diseased vessels, pre-PCI MLD). Because hs-CRP, eGFR and HbA1c were incompletely available, each is compared with the identical model fitted in exactly the same patients, so that any change in the RHI coefficient is attributable to the added covariate rather than to a change in sample composition. The RHI coefficient moved by no more than 0.03 for every covariate except smoking status, which attenuated it by 0.04, and no added covariate was itself associated with in-stent late lumen loss. Coefficients are not comparable across blocks, because each block is fitted in a different subset; the coefficient is larger in the smaller laboratory subsets than in the full cohort, which reflects the composition of those subsets rather than an effect of the covariates. p values are two-sided and derived from HC3 robust standard errors. †Nominal significance in this subset reflects its composition and does not apply to the full cohort. hs-CRP, high-sensitivity C-reactive protein; eGFR, estimated glomerular filtration rate; ACEi, angiotensin-converting-enzyme inhibitor; ARB, angiotensin-receptor blocker.

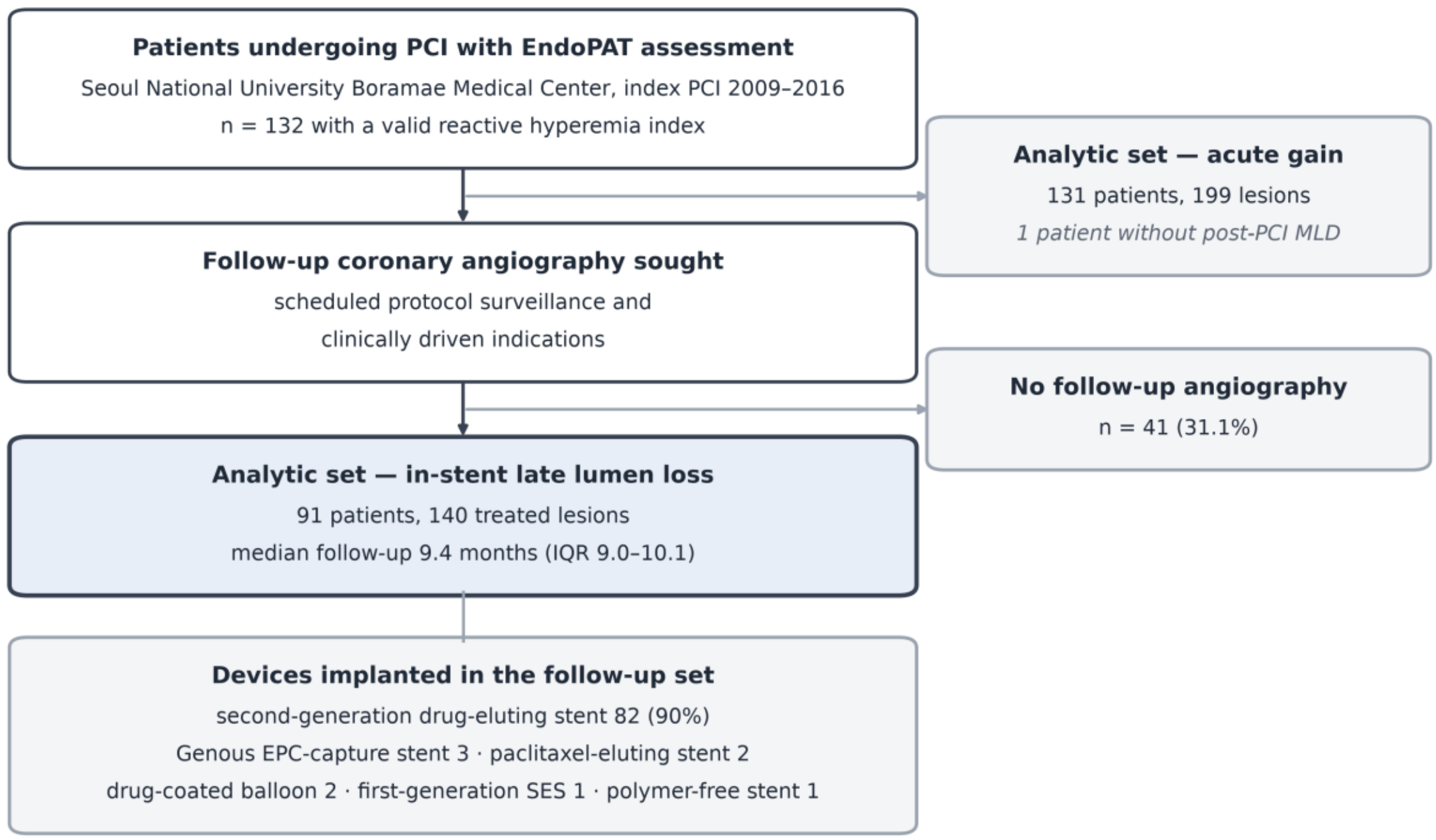

Supplementary Figure 1. Participant flow. Consecutive patients undergoing PCI with pre-procedural EndoPAT (n=132 with a valid RHI); follow-up coronary angiography (scheduled protocol surveillance plus clinically-driven indications) yielded the patient-level analytic set for in-stent late lumen loss (n=91; 140 lesions), with 41 patients not undergoing follow-up angiography. Device composition of the follow-up set is annotated; acute gain was analyzable in 131 patients.

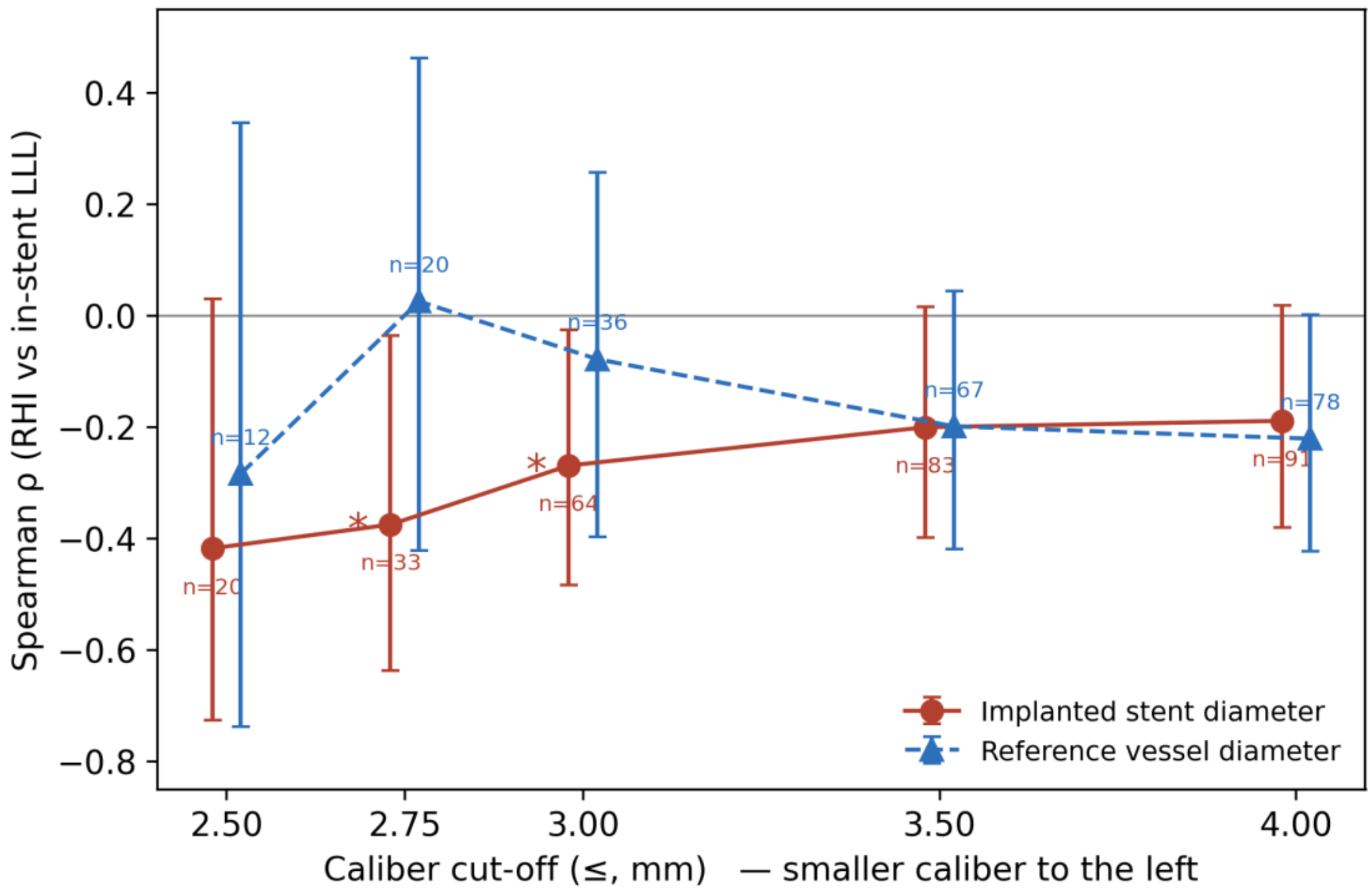

Supplementary Figure 2. [Exploratory, post hoc] Threshold-sensitivity of the RHI–in-stent LLL Spearman correlation across cumulative caliber cut-offs, for implanted stent diameter (circles, solid) versus angiographic reference vessel diameter (triangles, dashed). Points show ρ within each ≤cut-off subset; error bars, 95% confidence intervals; numbers, subgroup n; asterisks, nominal $p<0.05$. The inverse correlation was numerically stronger toward smaller implanted stent diameter (ρ=−0.19 overall to −0.42 at ≤2.5 mm) and was nominally significant at ≤2.75 and ≤3.0 mm, whereas reference vessel diameter did not show this small-caliber strengthening (≤2.75 mm, ρ=+0.03; ≤2.5 mm, ρ=−0.28 but n=12 with a wide confidence interval). Because the cut-off subsets are nested, adjacent points are not independent and the small-caliber estimates are imprecise; this exploratory display is hypothesis-generating.

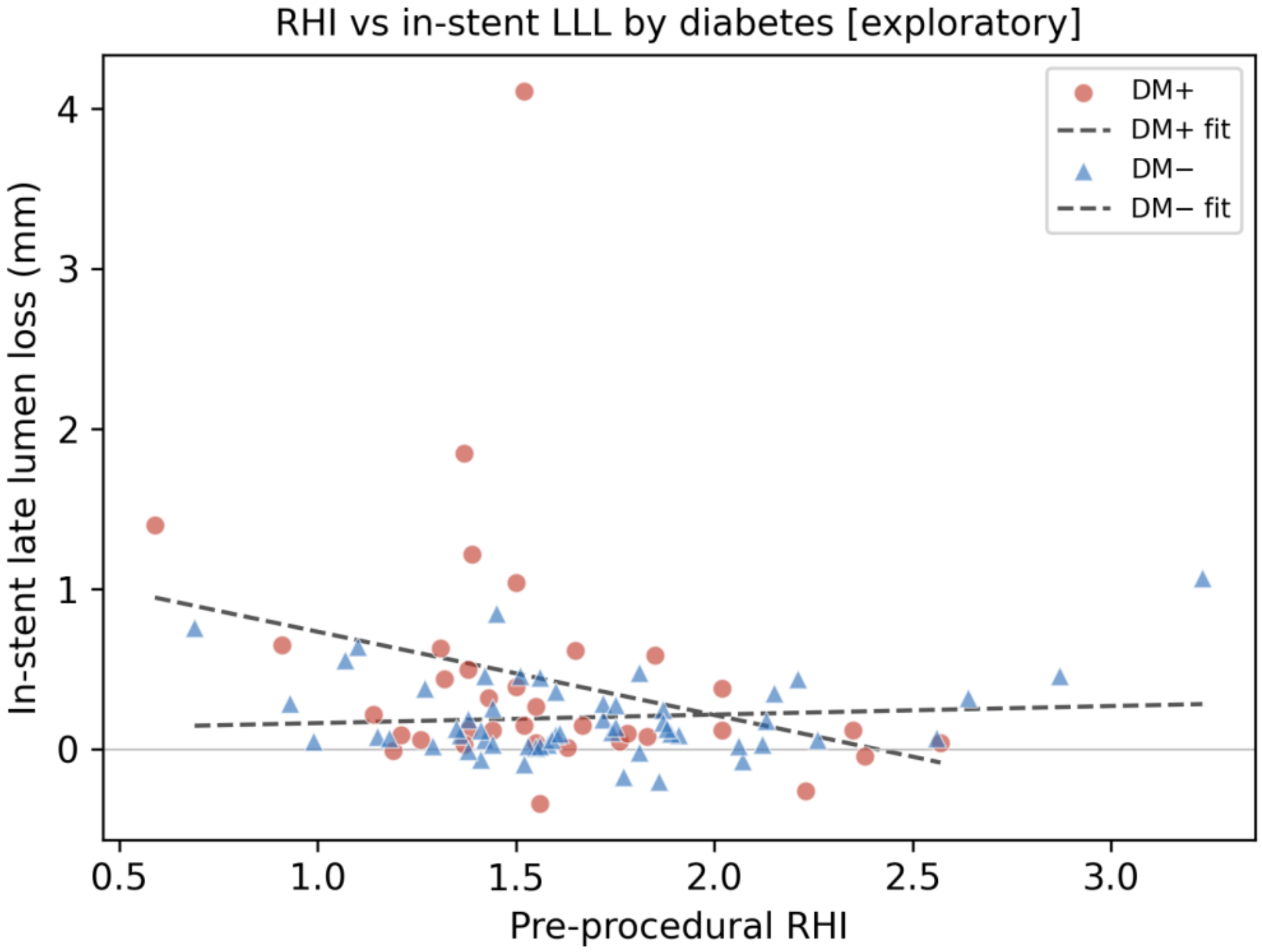

Supplementary Figure 3. [Exploratory] RHI versus in-stent late lumen loss by diabetes status. DM+ ρ=−0.37 (p=0.030, n=35); DM− ρ=−0.02 (p=0.88, n=56); the diabetes×RHI interaction was nominally significant in the primary covariate model (p=0.018) but not after additional adjustment for lesion length and smoking (p=0.35). x-axis, pre-procedural RHI.
